# Statistical methods to estimate the impact of remote teaching on university students’ performance

**DOI:** 10.1007/s11135-023-01612-z

**Published:** 2023-01-30

**Authors:** Silvia Bacci, Bruno Bertaccini, Simone Del Sarto, Leonardo Grilli, Carla Rampichini

**Affiliations:** 1 Department of Statistics, Computer Science, Applications “G. Parenti”, University of Florence (IT), Viale Morgagni 59, 50134 Florence, Italy; 2 Department of Political Science, University of Perugia (IT), Via Pascoli, 20, 06132, Perugia, Italy

**Keywords:** Difference-In-Differences, Distance learning, Higher education, Mixed model, Multilevel model

## Abstract

The COVID-19 pandemic manifested around the World since February 2020, leading to disruptive effects on many aspects of people social life. The suspension of face-to-face teaching activities in schools and universities was the first containment measure adopted by the Governments to deal with the spread of the virus. Remote teaching has been the emergency solution implemented by schools and universities to limit the damages of schools and universities closure to students’ learning. In this contribution we intend to suggest to policy makers and researchers how to assess the impact of emergency policies on remote learning in academia by analysing students’ careers. In particular, we exploit the quasi-experimental setting arising from the sudden implementation of remote teaching in the second semester of academic year 2019/2020: we compare the performance of the cohort 2019/2020, which represents the treatment group, with the performance of the cohort 2018/2019, which represents the control group. We distinguish the impact of remote teaching at two levels: degree program and single courses within a degree program. We suggest to use Difference-In-Differences approach in the former case and multilevel modeling in the latter one. The proposal is illustrated analysing administrative data referred to freshmen of cohorts 2018/2019 and 2019/2020 for a sample of degree programs of the University of Florence (Italy).

## Background, literature review and aims

Following the suggestion of the World Health Organization (WHO 2009), the closure of schools and universities is one of the primary measures included in many national plans aimed at containing a pandemic emergency. This is what happened during the COVID-19 pandemic when several policies to reduce social interactions were adopted by many Governments.

To mitigate the negative effects of an extended closure of schools and universities, the decision adopted by policy makers around the world was substantially unanimous: almost all teaching activities would be delivered online and remote teaching has become the most common answer to contain students’ loss of learning induced by the renunciation to face-to-face teaching. In Italy, the COVID-19 pandemic manifested since February 2020. The in-presence didactic activities of schools and universities were interrupted from the 5th of March until the end of the didactic period. For universities, the closure involved the second semester of the academic year 2019/20, including the examination sessions. See Crawford et al. (2020) for an overview of the measures adopted in 20 Countries in the higher educational field.

Schools and universities closure is far from a novelty. Such policies have already been locally applied in the recent past, after the epidemic events like the SARS (in 2003) and the A1/H1N1 (in 2009) outbreaks. Some early attempts to measure the impact of closures on possible loss of learning were made in relation to these two epidemics. Wong (2004) analysed the impact of e-learning during SARS outbreak in Hong Kong, finding evidence in favour of the online teaching as able to generate slightly better examination results than the traditional classes. After A1/H1N1 influenza, Goulas and Megalokonomou (2016) found in a sample of high school students from Greece that those who have the resources or the human capital to learn outside the classroom underperform when a strict attendance policy forces them to stay in the classroom.

Beyond these few isolated examples, it must be emphasised that closures had never been applied in such a pervasive way over such a long period, as happened from the beginning of the COVID-19 pandemic. The wide availability of studies arose during the COVID-19 pandemic on learning loss induced by online teaching attests the presence of a general concern about this issue (Cauchemez et al. 2009, 2014). The evolution of school closures across the world is measured by Unesco since 17 February 2020 (UNESCO 2022).

The sudden and pervasive passage from face-to-face teaching to remote teaching raised the issue about the consequences on the students’ learning processes and performances. It is worth to outline that the debate about remote learning compared to face-to-face learning started before COVID-19 emergency (see, among others, Arias et al. 2018). Both types of lessons delivery have pros and cons. On the one hand, remote teaching has several potential advantages. It is more cost-effective and it allows also students living in remote areas to have access to education. Moreover, in case of asynchronous lessons students can more easily manage their time to reconcile work, family, and study activities. But on the other hand, remote education strongly reduces collaboration and comparison among peers, as well as debate and discussion with teachers, and it requires more discipline and motivation from both students and teachers.

It should also be emphasised that remote teaching used to face COVID-19 pandemic was an *emergency* teaching differing from remote teaching implemented under “usual” conditions (Tuma et al. 2021). Iglesias-Pradas et al. (2021) note that the passage from face-to-face teaching to remote teaching usually takes six to nine months to be implemented and it requires to follow several steps (i.e., planning, preparation, development). In particular, teachers need to be trained to properly exploit technologies and resources, and to acquire pedagogical skills. Thus, classes offered around the world to mitigate the damages due to the closure of schools and universities should be labelled as emergency remote teaching.

The growing literature on the effects of emergency remote teaching on the university students’ performance is mainly based on specifically designed surveys (e.g., Aucejo et al. 2020), where students are asked about subjective evaluations of their university experience and performance with COVID-19 compared with the situation before COVID-19. These studies outlined numerous issues reported by university students (Dhahri et al. 2020; Fesol and Arshad 2020; Mahdy 2020; Rahiem 2021): a general difficulty to attend online lessons since curricula were not originally planned for online learning and teachers were not familiar with digital platforms; teachers’ lack of technological skills; teachers’ and students’ lack of suitable devices and stable and fast internet connection; difficulty to keep attention during the lesson; lack of private and exclusive spaces to attend lessons, and environmental features (e.g., lighting, noise, temperature) not specifically designed for learning (Realyvásquez-Vargas et al. 2020). Furthermore, university students reported an increased sense of anxiety and sleeping difficulties, a general loss of interest and motivation and difficulty in focusing on self-study, together with sense of loneliness and lack of effective communication and contact with other students and teachers. Additional issues are complained by students in medical fields about learning practical subjects (Prigoff et al. 2020; Sindiani et al. 2020; Wise et al. 2021). In brief, Rahiem (2021) outlined how staying at home and not being able to socialise with peers affect the mental well-being, generating stress and depression that can lead to a significant loss in learning and, hence, in academic performance.

Despite the problematic aspects of emergency remote teaching with respect to face-to-face teaching, the impact on academic students’ performance is not necessarily negative. Thus it is worth to measure the effect of remote teaching on the performance of university students comparing the students of academic year 2019/20, who experimented remote teaching during the second semester, with students of past cohorts, who had a regular teaching experience. This is a natural experiment, exploited by some studies, where the cohort 2019/20 is the treatment group and past cohorts are the control groups.

For instance, Gonzalez et al. (2020) analysed a sample of students enrolled at the Universidad Autónoma de Madrid, finding a significant positive effect of COVID-19 confinement on students’ performance in terms of exam scores and passing rates. The authors argued that this is due to a general improvement in the capability of autonomous learning. Meeter et al. (2020) carried out a study on more than 50,000 Dutch students in Psychology, observing higher grades for students that had attended remotely with respect to students of the previous years that had attended face-to-face the same courses; this positive effect interacted with performance at pre-pandemic time, with students with lower performance showing the higher gain. Iglesias-Pradas et al. (2021) analysed more than forty courses of the degree program in Telecommunication Engineering in a Spanish university, finding a significant increase in the overall academic performance with respect to the pre-pandemic experience, independently of the online class size, the choice of synchronous and asynchronous delivery, and the type of virtual communication tools. Differently from these cited studies, Talsma et al. (2021) found that students’ grades recorded for a sample of first-year psychology students from the 2020 cohort were not significantly different from grades recorded for a similar sample from the 2019 cohort.

In line with the above studies, the present contribution evaluates the impact of remote teaching on student performances using administrative data. However, our contribution is peculiar for the adopted statistical methodology, which allows us to measure the effect of remote teaching while controlling for student characteristics.

Differently from other studies, we evaluate the remote teaching impact at two levels of aggregation: (i) an overall impact on the entire degree program and (ii) a specific impact on the single courses within a degree program. From a methodological point of view, we evaluate the overall impact by means of Difference-In-Differences, which is a widely used approach to estimate the causal effect of a treatment in observational studies by comparing the change for treated units with the change for control units (Abadie 2010). The peculiarity of our proposal is to consider two cohorts of students as treatment and control groups and to measure the change in productivity between the first and second semester. As for the impact on single courses, we specify a multilevel (mixed) model (Snijders and Bosker 2012) with exams at level 1 and students at level 2. This approach allows us to estimate the change in the probability of passing each exam between the two cohorts controlling for observed and unobserved student characteristics.

The availability of administrative data allows us an objective evaluation on the whole population on students, avoiding issues of under-coverage, subjective judgements and missing values. For illustrative purposes, the analysis is performed on the administrative data referred to freshmen of cohorts 2018 and 2019 of some degree programs of the University of Florence (Italy), however the proposed approach is general, and it can be easily adapted to other universities.

The paper is organised as follows. In Sect. 2 data are illustrated, with a focus on the distribution of the students’ characteristics and the definition of an indicator of the students’ performance. In Sect. 3 the overall impact of remote teaching on students’ performance is analysed at the degree program level, whereas the impact of remote teaching at course level is investigated in Sect. 4. Details on the methodological approaches are also provided in both these sessions. Section 5 discusses the main results in light of the literature. Section 6 concludes with some final remarks.

## Data

We consider data from the administrative archive of the University of Florence on students’ careers. The available dataset includes the following students’ background characteristics: sex (male, female), high school type (scientific, classical, technical, vocational, other) and high school grade (integer ranging from 60 to 100). These variables are collected by the administrative office at the enrolment. The dataset is updated with information on taken exams.

In order to study the effect of remote teaching on students’ performance, we focus on first year compulsory courses, thus we consider exams registered until September of the year after enrolment. Each course is offered in one of the two semesters of the academic year. The first semester starts from September to mid-December, and the second, from mid-February to mid-May. In particular, we pay attention to two cohorts of freshmen enrolled in academic years 2018/19 and 2019/20 (labelled as cohort 2018 and 2019, respectively). Cohort 2018 did not experience remote teaching at all; on the other hand, as restrictive measures due to COVID-19 pandemic were undertaken in Italy at the beginning of March 2020, cohort 2019 experienced remote teaching only as regards the courses of the second semester.

In the following, the first section summarises students’ characteristics by degree program. The second section defines students’ performance in terms of passed exams.

### Students’ characteristics by degree program

Before starting the quantitative analysis, we performed a qualitative evaluation of the bachelor’s degrees offered by the University of Florence. We carefully inspected the study plans, and the corresponding offered courses, considering both the syllabus and teachers. We selected those programs that remained mainly unchanged for the two academic years of interest, i.e. 2018/19 and 2019/20. This qualitative analysis led to the choice of the following five bachelor degree programs: *i*. Chemistry (CHEM, for short); *ii*. Industrial design (DESIGN); *iii*. Law (LAW); *iv*. Mechanical engineering (ENGIN); *v*. Psychology (PSY).

The final dataset includes 2,790 students, whose characteristics are summarised in Table 1. The largest degree programs are Psychology and Law, with 853 and 768 enrolled students, respectively, which correspond to more than 50% of the total students considered in this work. On the other hand, Chemistry is the smallest one with 210 enrolled students. As regards the cohort of enrolment, students are approximately equally split over 2018 and 2019, with a slight predominance of the latter (+98 students). Moreover, females are slightly more than males (56.8%), whereas most of the students come from scientific high schools (37.6%).

Furthermore, Table 1 also reports students characteristics by degree program and cohort. Looking at the descriptive statistics related to the total, no cohort-specific, enrolled students, as far as the gender composition is concerned, we notice a clear prevalence of female students in Psychology (more than 75%), while in Industrial design and Law female students are still predominant, but with a lower extent (around 65%). Male students are definitely more frequent in Mechanical engineering (more than 80%), while Chemistry enrolment is almost equally distributed over males and females, with a slight predominance of the former. Moreover, high school grade (ranging from 60 to 100 in Italy) is, on average, quietly constant around 80 over the degree programs, with students of Industrial design exhibiting the lowest average high school grade (77.5). High school type composition reveals that most of students enrolled in Chemistry and Mechanical engineering clearly got a scientific high school degree, while a consistent share of students with a degree obtained in other high schools is observed in Industrial design and Psychology (44.0 and 34.5%). Finally, most of the students enrolled in Law is almost equally distributed over the scientific, classical and other types of high school, hence with low shares of students coming from technical or vocational schools.

**Table 1:** Descriptive statistics of freshmen by degree program and cohort: number of enrolled students (*N*), % of female, high school (HS) grade (mean with standard deviation within parentheses) and type of high school degree (%)

Degree program	Cohort	*N*	% fem.	HS grade	HS type (%)				
					Scien.	Class.	Techn.	Vocat.	Other
CHEM	2018	98	43.9	83.0 (12.1)	51.0	14.3	20.4	3.1	11.2
	2019	112	46.4	79.3 (12.1)	43.8	13.4	31.2	2.7	8.9
	Total	210	45.2	81.0 (12.2)	47.1	13.8	26.2	2.9	10.0
DESIGN	2018	149	69.8	76.8 (10.0)	32.2	4.7	19.5	6.0	37.6
	2019	167	62.3	78.2 (11.2)	22.8	7.8	18.0	1.8	49.6
	Total	316	65.8	77.5 (10.7)	27.2	6.3	18.7	3.8	44.0
LAW	2018	347	66.0	79.5 (10.7)	27.4	29.7	13.3	4.2	25.4
	2019	421	67.0	79.0 (11.9)	26.8	23.3	17.3	3.3	29.3
	Total	768	66.5	79.2 (11.4)	27.1	26.2	15.5	3.8	27.4
ENGIN	2018	325	18.2	81.2 (11.4)	55.7	3.7	35.1	2.4	3.1
	2019	318	18.9	79.0 (11.3)	57.9	4.7	31.4	1.3	4.7
	Total	643	18.5	80.1 (11.4)	56.8	4.2	33.3	1.9	3.8
PSY	2018	427	73.1	80.5 (10.7)	36.3	15.0	8.4	4.7	35.6
	2019	426	79.6	79.2 (11.2)	32.2	16.4	16.2	1.9	33.3
	Total	853	76.3	79.8 (11.0)	34.2	15.7	12.3	3.3	34.5
Overall		2790	56.8	79.6 (11.3)	37.6	14.7	19.8	3.1	24.7

CHEM: Chemistry; DESIGN: Industrial design; LAW: Law; ENGIN; Mechanical engineering; PSY: Psychology

### Measures of students’ performance

In order to evaluate the students’ performance, information on passed exams is inspected. First of all, each degree program has its own course allocation in terms of lessons and subsequent exams. In this regard, Table 2 reports an overview of the study plan envisaged by the selected degree programs. As we can see, students analysed in this work have to face three or four exams in each semester, with a number of total European Credit Transfer and accumulation System (ECTS) credits per semester equal to 27 or 30. The only exception is represented by those enrolled in Mechanical engineering, as it envisages two 12-credit annual courses, which are included in the second semester, as the passed exams related to these two courses are registered during the exam sessions of the second semester.

**Table 2:** Summary of the first year study plan for each degree program: number of courses and total credits (ECTS) envisaged by semester

Degree program	First semester		Second semester	
	No. of courses	Total credits	No. of courses	Total credits
CHEM	3	27	4	30
DESIGN	4	30	4	30
LAW	3	27	3	30
ENGIN$^*$	3	18	4	39
PSY	3	27	4	30

^*^Second semester includes two 12-credit annual courses

First of all, a clear and valid definition is needed to classify the exams taken by students. In the Italian academic system, the student can freely decide to follow a course in the semester envisaged by the study plan and take the related exam closely behind in the same semester (recommended path), or he/she can postpone both the attendance of the lessons and the moment (semester and academic year) for taking the exam. Moreover, there is no limit in the number of times a student can attempt to pass an exam related to the same course.

In this regard, an exam referred to a specific course held in a particular semester is labelled as:

- “passed”: the student passes the exam during the session of the semester which the course belongs to. For example, if a course belongs to the first semester (lessons held in the first semester) and the student passes the exam during the first semester exam session, that exam is considered as passed;
- “not passed”: the student fails to pass the exam in the session of the semester which the course belongs to. Consequently, it is considered as not passed, even if it has been passed in a subsequent session.

In the available data the proportion of passed exams is 54.9% in the first semester and 49.6% in the second one.

Finally, in order to define an overall indicator of student performance, we consider the proportion of gained credits, namely the sum of credits of passed exams divided by the expected sum of credits in the semester. In the available data the average proportion of gained credits is 0.556 (s.d. 0.379) in the first semester and 0.510 (s.d. 0.397) in the second one.

## Overall impact of remote teaching

The first part of the analysis focuses on the overall impact of remote teaching on each degree program. To this aim, we implement the Difference-In-Differences approach. In the following, we first describe the method, then we illustrate the results.

### The Difference-In-Differences approach

To evaluate the overall impact of remote teaching on student’s performance, separately for each degree program, we compare the number of ECTS credits gained during the second semester by the cohort 2018 and the cohort 2019, using information from the first semester to remove a possible “cohort effect” not depending on the remote teaching.

The idea is to compare the two selected cohorts relying on the Difference-In-Differences (DID) framework (Abadie 2010), considering the remote teaching as the treatment to be evaluated. In particular, the cohort 2018 did not experience remote teaching at all, thus it serves as the control group. On the other hand, the cohort 2019 experienced remote teaching in the second semester, thus it can be considered as the treated group. Moreover, the first semester, where none of the cohorts experienced remote teaching, is the pre-treatment period. On the other hand, the second semester, where only cohort 2019 experienced remote teaching, is the post-treatment period.

The performance of each cohort is measured by the average proportion of credits gained during the semester. We denote by ${\bar{Z}}_t^c$ the *average proportion of gained credits* in semester *t* for cohort *c*, with $t = 1, 2$ and $c = 0, 1$. In order to obtain the DID estimator, we consider the following four averages:

- ${\bar{Z}}_1^0$ for cohort 2018 (control group, $c = 0$) in the first semester (pre-treatment period, $t = 1$);
- ${\bar{Z}}_2^0$ for cohort 2018 ($c = 0$) in the second semester (post-treatment period, $t = 2$);
- ${\bar{Z}}_1^1$ for cohort 2019 (treated group, $c = 1$) in the first semester ($t = 1$);
- ${\bar{Z}}_2^1$ for cohort 2019 ($c = 1$) in the second semester ($t = 2$).

The DID estimate of the overall impact of remote teaching on student performance is therefore given by:

1 $$\begin{aligned} \text {DID} = ({\bar{Z}}_2^1 - {\bar{Z}}_1^1) - ({\bar{Z}}_2^0 - {\bar{Z}}_1^0). \end{aligned}$$

Estimator (1) is unbiased under the common trend assumption (Lechner 2011), that is, in absence of remote teaching the difference between gained credits in the two semesters would be identical for the two cohorts.

In order to get standard errors for inference, we implement the DID approach by means of a linear model with a fixed effect for each student (Angrist and Pischke 2008).

Let $Z_{it}$ be the proportion of credits gained by student *i* in semester *t*, with $t=1,2$ and $i=1,\ldots ,N_d$, where $N_d$ is the number of students enrolled in degree program *d*. The fixed effects model is

2 $$\begin{aligned} Z_{it} = \alpha _i + \gamma S_t + \delta C_{i} S_t + \varepsilon _{it}. \end{aligned}$$

Here, $\alpha _i$ is the fixed effect of student *i* summarising the effect of student’s observed and unobserved characteristics. The two dummy variables $C_i$ and $S_t$ represent the student cohort and the semester, respectively. Specifically, $C_i=1$ if student *i* belongs to the treatment group (i.e., cohort 2019) and $S_t=1$ if $t=2$ (i.e., second semester or post-treatment period). The main cohort effect $C_i$ is not included in the model equation, since it is collinear with the fixed effects $\alpha _i$. The interaction $C_{i} S_t$ equals 1 for the second semester of the treatment group, thus its coefficient $\delta$ represents the treatment effect. Finally, $\varepsilon _{it}$ is a zero mean residual.

Note that model (2) assumes a constant semester effect $\gamma$, corresponding to the common trend assumption required for identifiability of the treatment effect. In our setting, this assumption seems reliable. Indeed, the main characteristics of the students belonging to the two cohorts are unchanged, as reported in Table 1. Moreover, both cohorts enrolled in a pre-Covid period without any relevant change in the Italian educational system.

### Results at degree course program level

Table 3 reports the average proportions of gained credits out of the total envisaged credits by semester and cohort for each degree program. These data allow us to compute the DID estimate (1) of the overall remote teaching effect.

**Table 3:** Average proportion of credits gained by students of the different programs during the first year of study, by semester and cohort (2018 and 2019)

	CHEM		DESIGN		LAW		ENGIN		PSY	
Semester	2018	2019	2018	2019	2018	2019	2018	2019	2018	2019
First	0.357	0.281	0.565	0.462	0.631	0.642	0.516	0.488	0.579	0.618
Second	0.267	0.204	0.545	0.503	0.516	0.501	0.332	0.300	0.730	0.712
Difference	−0.090	−0.077	−0.020	0.041	−0.115	−0.141	−0.184	−0.188	0.151	0.094
DID	0.013		0.061		−0.026		−0.004		−0.057	

Looking at Table 3, we observe that the overall remote teaching effect depends on the degree program. In particular, for the Industrial design program we estimate an increase of about 6% in the number of acquired credits in the second semester under the remote teaching. On the contrary, for the Psychology program we observe a reduction of about 6%. For the other degree course programs the estimated effect is small.

In order to make inference on remote teaching effects, we fit model (2). Model fitting is performed separately for each degree program using the lm command of R (Chambers and Hastie 2017; R Core Team 2021). Model results are reported in Table 4.

**Table 4:** Estimates of regression model (2) for each degree program ($95\%$ confidence interval within parentheses)

Parameter	CHEM	DESIGN	LAW	ENGIN	PSY
Semester $$\gamma$$	−0.090$$^{***}$$	−0.020	−0.115$$^{***}$$	−0.184$$^{***}$$	0.151$$^{***}$$
	(−0.124; −0.056)	(−0.064; 0.024)	(−0.145; −0.084)	(−0.217; −0.151)	(0.121; 0.180)
Remote teaching $$\delta$$	0.013	0.061$$^\cdot$$	−0.026	−0.004	−0.056$$^{**}$$
	(−0.034; 0.059)	(−0.000; 0.122)	(−0.068; 0.015)	(−0.051; 0.042)	(−0.098; −0.015)
Error variance $\sigma ^2_\varepsilon$	0.015	0.038	0.042	0.045	0.048

***Significant at 0.001; **Significant at 0.01; *Significant at 0.05; $^\cdot$Significant at 0.10

As expected, the remote teaching effects $\delta$ estimated by the model are nearly equal to the DID estimates of Table 3. The confidence interval for Psychology does not include the zero, thus there is a significant negative effect of remote teaching on student performance in the second semester. On the contrary, for Industrial design there is a positive effect of remote teaching, but with a larger confidence interval, due to a smaller number of students. Nevertheless there is enough evidence of an effect, since the confidence interval is quite all on positive values. For the other degree programs there is no evidence of a significant effect of remote teaching on student performance.

It is worth to note that the negative effect for Psychology and the positive effect for Industrial design are confirmed, in the same data, by Carcaiso and Grilli (2022) who modelled the number of gained credits in the second semester by means of quantile regression for counts.

## Course-specific impact of remote teaching

The second part of the analysis evaluates the impact of remote teaching on student performance specifically for each course within the corresponding degree program. To this aim, we implement a generalised linear mixed modelling approach. In the following, we first specify the model, then, we illustrate the results.

### Generalised linear mixed modelling

The analysis presented in the previous section allows us to assess the overall impact of remote teaching at the degree program level. The results reported in Table 4 show a statistically significant impact for two out of the five degree programs. However, this approach does not take into account the heterogeneity of the courses within a given degree program.

In order to disentangle the impact of remote teaching at the course level, we compare the cohorts 2018 and 2019 in terms of passing the exams of each course of the second semester, adjusting for observed and unobserved student’s characteristics. In particular, the performance at the first semester is included in the model as a control variable to adjust for possible differences among students of the two cohorts.

Let $Y_{ij}$ be a binary variable, equal to 1 if student *i* (level 2) passes exam *j* (level 1), and 0 otherwise, with $i=1,\ldots ,N_d$ and $j=1,\ldots ,J_d$, where $J_d$ is the number of courses held in the second semester within degree program *d*. To take into account the correlation between exams of the same student, the probability of passing exam *j* by student *i*, $P(Y_{ij}=1)$, is modelled by means of a random intercept logit model:

3 $$\begin{aligned} \text {logit}\bigl [ P(Y_{ij}=1 \mid {\varvec{{x}}}_i, C_i) \bigr ] = \gamma _{j} + \delta _j C_i + {\varvec{{x}}}_i' {\varvec{\beta }} + u_i, \end{aligned}$$

where ${\varvec{{x}}}_i$ is the vector of student covariates and $C_i$ is a dummy variable denoting the cohort ($C_i=1$ if student *i* belongs to cohort 2019). Random effects $u_i$ are independent normally distributed, with zero mean and constant variance $\sigma _u^2$. Model (3) has a specific exam intercept $\gamma _j$, which is the logit of the probability of passing exam *j* for a student of cohort 2018 with baseline values, that is, ${\varvec{{x}}}_i=0$ and $u_i=0$. Parameter $\delta _j$ is the change in the model intercept between the two cohorts, namely the difference of the logit of passing exam *j* between cohort 2019 and cohort 2018. This parameter therefore summarises the effect of remote teaching on exam *j*.

### Results at course level

The random intercept logit model (3) is fitted separately for each degree program using the glmer command included in the R package lme4 (Bates et al. 2015, 2021). For Industrial design, we have excluded a course with a zero passing rate in 2019 (see Table A1 in the Appendix). The estimated coefficients are reported in the Appendix (Tables A2 and A3).

Before looking at the model estimates, a likelihood ratio test is performed to justify the choice of a multilevel approach by comparing the two-level logit model with the single-level one (i.e. $\sigma ^2_u=0$). The test is significant for all degree programs, with *p*-value $=0.0473$ for Chemistry and $p<0.001$ for the others. Consequently, we can state that $\sigma _u^2$ is significantly different from 0; hence a mixed modelling approach is suitable for modelling the data at issue. Moreover, we prefer to specify a random effects model because the fixed-effects model would not allow us to include covariates at the student level (level 2 variables). Furthermore, as the fixed-effect specification requires conditional maximum likelihood estimation, we would need to remove from the analysis the students who passed all the scheduled exams and those who failed all the exams.

The fitted model adjusts for student performance in the first semester by means of two covariates, namely the proportion of gained credits during the first semester and a dummy variable for students getting zero credits during the first semester. Such non linear specification allows us to account for students who did not start taking exams during the first semester. The two covariates of student’s performance account for a relevant part of student level variability: as an example, for Psychology the standard deviation of the random effects, $\sigma _u$, reduces from 4.068 to 2.704. Conditionally on student’s performance, the other student characteristics (gender, high school grade and type) are not significant, thus they are not included in the estimated model.

The effect of the remote teaching on exam *j* is measured on the logit scale by the parameter $\delta _j$ of Eq. (3). To get an interpretation in terms of probability, we compute the average marginal effect (AME, Agresti and Tarantola 2018), namely the average discrete change in the probability of passing the exam between 2018 and 2019. Table 5 reports the estimated AMEs by program degree, computed using commands melogit and margins in Stata. For example, the first course of Chemistry (CHEM01 in the table) has an AME equal to $-0.066$, meaning an average reduction of $6.6\%$ in the passing probability.

**Table 5:** Average marginal effects (AME) and standard errors (SE) by degree program from GLMM of Eq. (3)

Course	AME	SE
CHEM		
CHEM01	−0.066${^{\cdot }}$	0.035
CHEM02	−0.034	0.028
CHEM03	0.079${^{\cdot }}$	0.042
CHEM04	0.017	0.033
DESIGN		
DES01	0.064	0.046
DES02	0.066${^{\cdot }}$	0.038
DES03	0.013	0.041
LAW		
LAW01	−0.053${^{\cdot }}$	0.028
LAW02	0.036	0.028
LAW03	−0.046	0.028
ENGIN		
ENG01	0.096$$^{**}$$	0.029
ENG02	0.108$$^{**}$$	0.033
ENG03	−0.167$$^{***}$$	0.029
ENG04	0.010	0.032
PSY		
PSY01	−0.082$$^{**}$$	0.026
PSY02	−0.017	0.025
PSY03	−0.086$$^{***}$$	0.025
PSY04	0.025	0.022

$${^{{***}}}$$Significant at 0.001; $$^{{**}}$$Significant at 0.01; $$^{{*}}$$Significant at 0.05; $${^{\cdot }}$$Significant at 0.10

It is not possible to draw a general conclusion on the impact of remote teaching on the probability of passing exams. Indeed, in each degree program there are both courses with positive effect and courses with negative effect. In most cases, the effects are not statistically significant. However, there are some courses with noteworthy effects that need further investigation to understand the reasons of such changes. In particular, courses ENG01 and ENG02 report a statistically significant increase of about 10% of passing probability and ENG03 is associated with a significant reduction of more than 16%: the overall impact on the degree program ENGIN is null, as outlined by the not significant $\delta$ coefficient estimated in the DID analysis (Table 4 in Sect. 3.2). Differently, for two out of the four courses of degree program PSY, that is, PSY01 and PSY03, the passing probability significantly reduces more than 8% with respect to the pre-Covid period, leading to an overall significantly negative impact of remote teaching on the degree program with an average reduction of the number of acquired credits of 5.6% (Table 4).

## Discussion

During the first phase of the COVID-19 pandemic, remote teaching was an emergency strategy adopted all around the World by universities. Due to the emergency, it was implemented in different ways, not only among different universities, but also within a same university: indeed, even if there were broad guidelines at university level, every teacher took specific actions. Therefore, the impact on student performance is quite heterogeneous. Indeed, our analysis at the University of Florence revealed both positive and negative effects among degree programs and within courses of a given degree program, even if in most courses the effect is not statistically significant. Our results point out that the effect of remote teaching is more heterogeneous that what found in previous studies carried out on administrative data. Indeed, such studies agreed on a significant positive increase in the overall academic performances (Gonzalez et al. 2020; Meeter et al. 2020; Iglesias-Pradas et al. 2021), with the only exception of Talsma et al. (2021), where no significant difference were found between students from the 2020 cohort and students from the 2019 cohort.

It should be noted how our study, together with the other ones based on careers data, reveals a definitely less negative impact of remote teaching on student performances than what suggested by recent studies based on surveys (Aucejo et al. 2020; Dhahri et al. 2020; Fesol and Arshad 2020; Mahdy 2020; Rahiem 2021). These last studies investigated on students’ subjective evaluations, motivation, satisfaction, and feeling about remote teaching during COVID-19 pandemic, raising considerable and unanimous critical issues. However, in spite of negative perceptions and substantial dissatisfaction, the objective results in terms of performance show a general capability of students to face an emergency situation without incurring significant arrests in the university career. Along these lines, Biwer et al. (2021) outlined the general capability of the majority of students to compensate challenges due to remote teaching: after having clustered university students into four profiles of adaptation (overwhelmed, surrenderers, maintainers, adapters), they find that only the surrenderers, corresponding to about one fourth of the students, show a decreased investment of time and effort in the self-study, while the remaining show an increase or no significant change.

A possible framework to interpret the observed results is provided by the Study Demands-Resources (SD-R) theory (Lesener et al. 2020) that examines the relationships between study characteristics and study performance. Applying the same principles of the Job Demands-Resources theory (Demerouti et al. 2001) developed in the job setting to explain the workers’ burnout and well-being, the SD-R theory detects study demands (e.g., attending lectures, investing time in self-studying, managing high workload), which have a positive impact on the student burnout, and study resources (e.g., support from teachers, self-efficacy, self-motivation), which have a negative effect on the student burnout and a positive effect on the student engagement; in turn, student burnout and student engagement contribute in negative way and in a positive way, respectively, to the student performance.

To take into account the consequences of COVID-19 on teaching and learning, the SD-R theory can be extended with new demands and resources under the remote teaching setting (Martin et al. 2021). On one hand, among the new demands we can detect at least three types of challenges (Aguilera-Hermida 2020): environmental (e.g., difficulty to be concentrated while being at home), emotional (e.g., lack of motivation), and online educational (e.g., increased workload) challenges, among which the techno-stressors (e.g., online learning barriers, techno-overload, work-home conflict, techno-ease, techno-reliability, techno-sociality; Galvin et al. 2022) play an important role. On the other hand, among the new study resources induced by the remote teaching, we can cite lectures registrations, increased time for family and hobbies, major stimulus for autonomy and adaptability, online support from university/teachers, parent help.

According to the SD-R theory, the substantial neutral effect on the study performance observed at the University of Florence can be explained with a compensation between the new study demands and the new study resources; see also Talsma et al. (2021) (and references therein) for a similar interpretation.

## Final remarks

The aim of this contribution is to provide some guidelines to help researchers and academic policy makers to assess the effect of remote teaching on university students’ performance.

At a first level of analysis, we suggest to consider the impact on each degree program, exploiting the quasi-experimental setting arising from the sudden implementation of remote teaching. To this end, we rely on the Difference-In-Differences approach, which is based on a common trend assumption that is reasonable in this setting. Moreover, this approach is straightforward.

As the overall effect for a given degree program may arise from heterogeneous effects on single courses, at a second level of analysis we suggest to focus on the performance at the corresponding exams. The proposed approach, via a random intercept logit model, allows us to estimate the effect of remote teaching on each course adjusting for observed and unobserved student characteristics. If available, course characteristics can be included as covariates in the model to explain the heterogeneous course effects.

Our analysis has two limitations due to the nature of available data. First, we considered outcomes based on exams results. However, passing an exam is just a proxy of learning achievement. Second, our data does not contain information on exam type, which in most cases changed substantially (e.g., an essay converted into a quiz) and, more generally, on the specific policies adopted by teachers (e.g., more flexibility in evaluating the students’ preparation). It is not thus possible to disentangle the effect of remote teaching itself from the effect of new exam rules and teacher attitude towards students. This suggests to be cautious in comparing the outcomes across courses. Despite these limitations, the proposed approach allowed us to estimate a total effect of remote teaching on student performance, which is valuable in order to highlight programs and courses with noteworthy changes to be inspected by the university staff.

## Appendix

See Tables A1, A2, A3.

**Table A1:** Percentage of students that passed the exams envisaged in the second semester of the first year of each degree program: comparison of observed raw outcomes between cohorts

		Cohort		
Credits	Course	2018	2019	Diff.
CHEM				
6	CHEM01	11.2	5.4	−5.8
12	CHEM02	42.9	29.5	−13.4
6	CHEM03	9.2	14.3	5.1
6	CHEM04	27.6	23.2	−4.4
DESIGN				
6	DES01	80.5	77.2	−3.3
6	DES02	61.1	60.5	−0.6
12	DES03	63.8	56.9	−6.9
6	DES04	3.4	0.0	−3.4
LAW				
12	LAW01	57.3	53.0	−4.3
9	LAW02	36.9	41.3	4.4
9	LAW03	58.8	55.1	−3.7
ENGIN				
9	ENG01	47.1	57.2	10.1
6	ENG02	14.8	22.0	7.2
12	ENG03	44.0	23.6	−20.4
12	ENG04	21.2	20.1	−1.1
PSY				
9	PSY01	77.5	72.1	−5.4
9	PSY02	75.4	76.1	0.7
6	PSY03	69.6	62.7	−6.9
6	PSY04	66.0	71.4	5.4

**Table A2:** GLMM estimates with standard errors (SE) of model (3): Chemistry, Industrial design and Law

Coefficient	Estimate	SE
Chemistry (CHEM)		
*Student covariates* (${\varvec{\beta }}$)		
Proportions of gained credits in semester I	9.502$$^{***}$$	1.304
No exam in semester I	0.233	0.875
*Exam parameters cohort 2018* ($\gamma _j$)		
CHEM01	−3.981$$^{***}$$	0.641
CHEM02	0.739$^\cdot$	0.415
CHEM03	−4.391$$^{***}$$	0.689
CHEM04	−1.396$$^{**}$$	0.446
*Exam differences 2019 vs 2018* ($\delta _j$)		
CHEM01	−1.335$^\cdot$	0.772
CHEM02	−0.656	0.575
CHEM03	1.390$^{*}$	0.696
CHEM04	0.311	0.603
Standard deviation of random effects ($\sigma _u$)	0.897$^{*}$	0.363
Industrial design (DESIGN)		
*Student covariates* (${\varvec{\beta }}$)		
Proportions of gained credits in semester I	3.977$$^{***}$$	0.645
No exam in semester I	−3.266$$^{***}$$	0.663
*Exam parameters cohort 2018* ($\gamma _j$)		
DES01	2.281$$^{***}$$	0.344
DES02	0.595$^{*}$	0.268
DES03	0.794$$^{**}$$	0.272
*Exam differences 2019 vs 2018* ($\delta _j$)		
DES01	0.591	0.446
DES02	0.607	0.372
DES03	0.118	0.369
Standard deviation of random effects ($$\sigma _u$$)	1.554$$^{***}$$	0.218
Law (LAW)		
*Student covariates* (${\varvec{\beta }}$)		
Proportions of gained credits in semester I	6.854$$^{***}$$	0.471
No exam in semester I	0.830$^{*}$	0.406
*Exam parameters cohort 2018* ($\gamma _j$)		
LAW01	−0.479$^{*}$	0.200
LAW02	−2.219$$^{***}$$	0.225
LAW03	−0.352$^\cdot$	0.200
*Exam differences 2019 vs 2018* ($\delta _j$)		
LAW01	−0.458$^\cdot$	0.245
LAW02	0.313	0.244
LAW03	−0.403	0.245
Standard deviation of random effects ($$\sigma _u$$)	1.471$$^{***}$$	0.136

$$^{***}$$Significant at 0.001; $$^{**}$$Significant at 0.01; $$^{*}$$Significant at 0.05; $$^\cdot$$Significant at 0.10

**Table A3:** GLMM estimates with standard errors (SE) of model (3): Engineering and Psychology

Coefficient	Estimate	SE
Engineering (ENGIN)		
*Student covariates* (${\varvec{\beta }}$)		
Proportions of gained credits in semester I	3.110$$^{***}$$	0.320
No exam in semester I	−1.165$$^{***}$$	0.309
*Exam parameters cohort 2018* ($\gamma _j$)		
ENG01	−0.027	0.172
ENG02	−2.516$$^{***}$$	0.224
ENG03	−0.236	0.172
ENG04	−1.903$$^{***}$$	0.201
*Exam differences 2019 vs 2018* ($\delta _j$)		
ENG01	0.754$$^{***}$$	0.225
ENG02	0.846$$^{**}$$	0.264
ENG03	−1.308$$^{***}$$	0.237
ENG04	0.074	0.252
Standard deviation of random effects ($$\sigma _u$$)	1.068$$^{***}$$	0.106
Psychology (PSY)		
*Student covariates* (${\varvec{\beta }}$)		
Proportions of gained credits in semester I	7.386$$^{***}$$	0.660
No exam in semester I	−0.063	0.518
*Exam parameters cohort 2018* ($\gamma _j$)		
PSY01	2.794$$^{***}$$	0.323
PSY02	2.476$$^{***}$$	0.315
PSY03	1.660$$^{***}$$	0.298
PSY04	1.205$$^{***}$$	0.290
*Exam differences 2019 vs 2018* ($\delta _j$)		
PSY01	−1.132$$^{**}$$	0.349
PSY02	−0.243	0.349
PSY03	−1.190$$^{***}$$	0.326
PSY04	0.361	0.329
Standard deviation of random effects ($$\sigma _u$$)	2.704$$^{***}$$	0.204

$$^{***}$$Significant at 0.001; $$^{**}$$Significant at 0.01; $$^{*}$$Significant at 0.05; $$^\cdot$$Significant at 0.10
